# Draft genome sequences of *Bacillus benzoevorans* DSM 5391^T^ and *Bacillus oleivorans* JC228

**DOI:** 10.1128/mra.00719-26

**Published:** 2026-10-07

**Authors:** Imade Y. Nsa, Qowiyyat A. Adeniyi, Oluwafoyinsolami I. Adisa, Samuel O. Mojirayo, Deborah O. Oyekunle, Emmanuel T. Ajayi, Rüdiger Pukall, Alex Spunde, Supratim Mukherjee, Markus Göker, Rekha Seshadri

**Affiliations:** 1 Department of Microbiology, Faculty of Life Sciences, University of Lagos 70670, Lagos, Nigeria; 2 Leibniz Institute DSMZ—German Collection of Microorganisms and Cell Cultures GmbH, Braunschweig, Germany; 3 DOE Joint Genome Institute, Lawrence Berkeley National Laboratory 1666, Berkeley, California, USA; University of Wisconsin-Madison, Madison, Wisconsin, USA

**Keywords:** hydrocarbon-degrading bacteria

## Abstract

We present the genome sequences of two hydrocarbonoclastic *Bacillus* spp. type strains—*Bacillus benzoevorans* DSM 5391^T^ isolated from soil in Marseille, France (4.7 Mb: 40.04% guanine–cytosine [GC]) and *Bacillus oleivorans* JC228 (4.5 Mb: 39.16% GC)—isolated from a contaminated Petri dish in Hyderabad, India.

## ANNOUNCEMENT

Bacilli are versatile, growing on nutrient sources derived from natural and synthetic compounds and yielding numerous products (1). *Bacillus benzoevorans* DSM5391^T^, a facultative anaerobe (2), was isolated from soil in Marseille, France (3), while *Bacillus oleivorans* JC228, an obligate aerobe, was isolated from a contaminated laboratory plate in Hyderabad, India (4). These gram-positive rods are hydrocarbonoclastic. *B. benzoevorans*, identified based on its nutritional and metabolic features, is non-saccharolytic, utilizing phenols and various carboxylic acids as major carbon and energy sources (3). *B. oleivorans* is capable of utilizing diesel oil as its sole carbon, tolerates toxic solvents, and is identified based on the 16S rRNA sequence identity (4).

The genomes were sequenced as part of the Department of Energy (DOE) Joint Genome Institute Genomic Encyclopedia of Bacteria and Archaea projects (5). *B. benzoevorans* DSM 5391^T^ was grown on DSMZ Medium 1 supplemented with 0.4% yeast extract and 0.2% benzoate and incubated at 30°C for 2 days (4). The genomic DNA was extracted using the MasterPure Gram-Positive Extraction Kit (Epicenter, WI, USA), while *B. oleivorans* was maintained on nutrient agar. For both strains, 200 ng of DNA was sheared to ~300 bp, and fragments underwent end repair, A-tailing, and adapter ligation (IDT, USA) using the KAPA-Illumina Library Kit (KAPA, USA). For both genomes, libraries were prepared for sequencing following a 2 × 150 bp indexed run. The Illumina NovaSeq 6000 platform using NovaSeq XP v1 reagent kits and an S4 flow cell generated 8,613,532 reads totaling 1,300,643,332 bp (*B*. *benzoevorans*), and Illumina HiSeq 2500 generated 7,063,540 reads totaling 1,066,594,540 bp (*B. oleivorans*).

**Fig 1:**
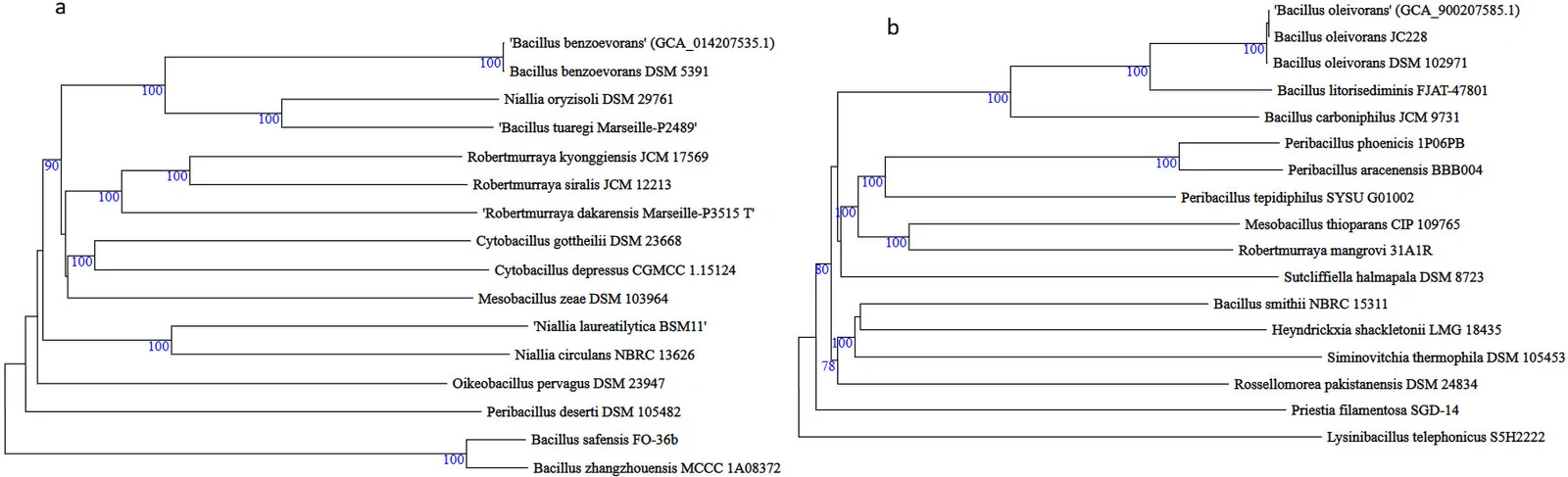
Phylogenomic tree of strains based on whole proteome similarity with type strains using the Type Strain Genome Server (TYGS) (**a**) *Bacillus benzoevorans* and (**b**) *Bacillus oleivorans.*

Raw paired-end reads were filtered using BBTools v38.76 (*B*. *benzoevorans* DSM 5391^T^) and v37.17 (for JC228) (6), then assembled with SPAdes v3.13.0 and v3.10.1, respectively (7). Next, 200 bp was trimmed from contig ends; contigs shorter than 1 kb and with a coverage <2 were discarded using BBTools. The final assemblies consisted of 63 contigs in 55 scaffolds, totaling 4,701,532 bp (*B. benzoevorans*), and 27 contigs in 26 scaffolds, totaling 4,511,824 bp (*B. oleivorans*), and genome coverage of 273× and 230×, respectively. The taxonomy of the strains was verified using a whole proteome-based tree on TYGS (8–10) (Fig. 1). Both genomes were annotated using the IMG annotation pipeline, which predicted 4,639 genes, 140 RNAs for *B. benzoevoran*s (v5.0.12), and 4,445 genes and 113 RNAs for *B. oleivorans* (v4.15.1) (11) (Table 1). Default parameters were used for all software.

**TABLE 1:** *De novo* assembly and genome features of *Bacillus benzoevorans* DSM 5391^T^ and *Bacillus oleivorans* JC228

Property	*Bacillus benzoevorans* DSM 5391^T^	*Bacillus oleivorans* JC228
Total scaffold sequence length (bp)	4,701,532	4,511,824
Number of contigs	55	26
Contig N50 (kb)	198.6	470
Average fold coverage (×)	273.0	230
GC content (%)	40.04	39.16
Total predicted genes	4,866	4,558
Predicted protein-coding genes	4,639	4,445
rRNA genes	27	15
5S rRNA	11	5
16S rRNA	12	5
23S rRNA	4	5
Other RNA genes	9	22
tRNA genes	104	76
CheckM2 completeness	100	100
CheckM2 contamination	1.63	1.09

Genome annotation of *B. benzoevorans* predicted a reduced phosphotransferase system (PTS) and expanded multiple copies of 3-hydroxybutyryl CoA-dehydrogenase and oxygenases, including a long-chain alkane monooxygenase 2-hydroxymuconate-semialdehyde hydrolase, whereas *B. oleivorans* possesses a broad-spectrum PTS of diverse putative saccharolytic enzymes but fewer dehydrogenases and β-oxidation genes for metabolizing alcohol, aldehydes, and fatty acids associated with hydrocarbons. The presence of putative catechol 2,3-dioxygenases in both organisms suggests a meta cleavage pathway for biodegradation. The higher copy number of these genes supports *B. benzoevorans* as being metabolically primed for aromatic hydrocarbon degradation.

## Data Availability

The high-quality draft genome annotation of *Bacillus benzoevorans* DSM 5391^T^ and *Bacillus oleivorans* JC228 is available from the Joint Genome Institute IMG/M taxon IDs 2861430565 and 2740891863, respectively. This Whole Genome Shotgun project has been deposited in GenBank under accession numbers JACHGK000000000 and OAOP00000000; project data are available under BioProject accession numbers PRJNA632316 and PRJNA346210 with BioSample accession numbers SAMN14908537 and SAMN05877753 and Sequence Read Archive (SRA) SRX8733760 and SRX3161845 for *B. benzoevorans* and *B. oleivorans*, respectively.
